# PGF2alpha induced differential expression of genes involved in turnover of extracellular matrix in rat decidual cells

**DOI:** 10.1186/1477-7827-3-3

**Published:** 2005-01-11

**Authors:** Eduardo A Callegari, Susan Ferguson-Gottschall, Geula Gibori

**Affiliations:** 1 Department of Physiology and Biophysics, University of Illinois at Chicago, Chicago, Illinois, USA; 2 Biomedical Resources Infrastructure Network (BRIN), Division of Basic Biomedical Sciences, The University of South Dakota School of Medicine, 414 E. Clark St, Vermillion, SD 57069, USA

## Abstract

In the rat, the decidual tissue is an important component for maternal recognition of pregnancy. Decidualization can be induced by either the implantation of the blastocyst or by artificial stimuli. The process of decidua formation or decidualization, is characterized by growth and differentiation of endometrial stromal cells. Prostaglandin F2alpha (PGF2α) has been shown to be involved in inhibition of implantation, alteration of embryo development, induction of luteal regression, and the mediation of pregnancy loss induced by microorganism infections. In order to establish a direct role for PGF2α in decidual function, we have evaluated its effects on the expression of an extensive array of genes using primary decidual cell culture. Upon treatment with PGF2α sixty genes were significantly down-regulated whereas only six genes were up-regulated (from a total of 1176 genes studied). Interestingly, the majority of the genes inhibited by PGF2α are either directly or indirectly involved in the turnover of the extracellular matrix (ECM). Genes such as gelatinase A (MMP2), cathepsin L, tissue inhibitor metalloproteinases 2 (TIMP2) and 3 (TIMP3), plasminogen activator inhibitor1 (PAI1), tissue type plasminogen activator (tPA), urokinase plasminogen activator (tPA), endothelin 1, calponin, carboxypeptidase D and calponin acidic were down regulated. The opposite effect was observed for prostromelysin 53 kDa (proMMP3), plasma proteinase I alpha and alpha 1 antiproteinase, all of which were significantly up-regulated by PGF2α. The results strongly suggest that the abortificient role of elevated levels of PGF2α after implantation is due, in large part, to inhibition of genes involved in the normal turnover of the extracellular matrix necessary for decidual formation.

## Background

The establishment of successful pregnancy requires a profound reorganization of uterine tissues. Rapid growth and differentiation of the endometrial stroma is the earliest and most striking event in pregnancy. Differentiation of stromal cells leads to the formation of unique cells, termed decidual cells, which differ greatly from the original stromal cells [1]. The decidua is an important component in the maternal recognition of pregnancy and can be induced by either the implantation of the blastocyst or by artificial stimuli. An interesting feature is that – the growth and differentiation of the endometrial cells – occurs differently in different regions of the uterus [1]. Mesometrial decidual cells are formed after the antimesometrial decidua and their death takes place after antimesometrial cell degeneration. Regression of the decidual cells appears to be controlled by an intrinsic cell death program of apoptosis, which takes place after day 10 of pregnancy in the antimesometrial region first, followed by the mesometrial region [2].

Prostaglandin F_2α _can induce different biological actions at the beginning and at the end of pregnancy. PGF_2α_, Prostaglandin E_2 _(PGE_2_), and 6-keto-PGF_1α _are produced by the pregnant uterus [3]. An increase of uterine PGE_2 _and PGF_2α _is observed on day 5 of pregnancy, allowing the decidualization process to take place. When embryo access to the uterus is impaired, production of prostaglandins (PGE_2 _and PGF_2α_) is suppressed [4]. During postimplantation, PGE2 returns to the original preimplantation levels, but PGF_2α _decreases [4]. Whereas PGF2α contributes to the process of decidualization, implantation and recognition of pregnancy. An increase in PGF_2α _over certain values can terminate pregnancy [6]. A high level of PGF_2α _is known to induce inhibition of implantation, alteration of embryo development, and induction of luteal regression [5]. During infection, an inflammatory response mediated by cytokines can be generated [7]. This release of PGF_2α _can induce premature uterine contraction and premature labor [8]. Depending on which stage of pregnancy an increase of PGF_2α _secretion occurs, it can alter implantation, induce abortion or even embriolethality [7,8]. Because an increase in PGF_2α _levels after implantation can be detrimental for the progression of pregnancy, the aim of this investigation was to determine whether PGF_2α _affects directly decidual cells and leads to disturbance in the expression of genes crucial for decidual survival.

## Methods

### Animal model

Pseudopregnancy was induced by mating Holtzman Sprague Dawley female rats with vasectomized male rats. The day a vaginal plug was found was designated day 1 of pseudopregnancy. Decidualization of the uterine endometrium was induced by scratching the antimesometrial surface with a hooked needle on day 5 of pseudopregnancy under ether anaesthesia. Animals were housed in a controlled environment (22–24°C) and kept under controlled conditions (lights on; 0500–1900 hrs) with free access to standard rat chow and water. The University of Illinois at Chicago animal care and use committee approved the animal care and handling.

### Primary decidual cell culture and hormone treatment

For each experiment 15 pseudopregnant rats were used. Decidual cells were isolated as previously described [9]. Cells were pooled and seeded into six-well plates (1.4 × 10^6 ^cells/ well). They were allowed to attach 3–4 hrs before washing in PBS to remove erythrocytes. Cells were incubated in RPMI 1640 without phenol red (Mediatech, Whashington DC), containing 1% CD-FBS (HyClone Laboratories Inc, Logan, UT), and treated with or without high levels (5 μM [10,11]) of PFG_2α _(Sigma, St. Louis, MO) for 12 hr). After incubation, the cells were harvested in cold PBS, quick frozen in liquid nitrogen and kept at -80 C until RNA isolation.

### Gene identification by cDNA array

Total RNA was isolated from primary decidual cells (PDC) by using a RNAII isolation kit (Clontech, Palo Alto, CA), following the manufacture's instructions. RNA isolated from wells treated with either PGF_2α _or vehicle were pooled independently and subjected to cDNA array. cDNA probes were generated from 4 μg of total RNA in a reverse transcriptase reaction using a mix of dATP, dTTP and dGTP (Takara Biomedicals, Shiga, Japan) plus [α^32^P]-dCTP (Amersham, MO), and a mixture of primers specific to each gene present in the array. All probes used had 5 to 10 × 10^6 ^cpm and the difference between control and experimental probes in each assay was less than 10%. cDNA were hybridized to an Atlas Rat 1.2 Array (#7854-1) nylon membrane (Clontech, CA). Hybridization and post-hybridization washes were performed according to the manufacturer's protocol. The signal was scanned with a phosphorImager (Molecular Dynamics, CA) after 48 h of exposure. Control and experimental RNA were always processed in parallel.

### Data analysis

Spot intensities from scanned membranes were analyzed using the AtlasImage 1.5 software (Clontech, CA). Grids were orientated manually and adjusted to ensure optimal spot recognition using AtlasImage's fine tuning tools, discarding spots with dust or locally high background. The software makes the analysis for background correction and normalization versus housekeeping genes. It also calculate the ratio and indicates which genes has a ratio > 2 (up regulated), or ratio < 0.5 (down regulated) or 0.5 < ratio < 2 (equal expression). Gene expression data were normalized using the Sum method included in the Atlas Image software. Data points where the expression was not greater than two standard deviations were discarded. For the final analysis, data points were the averages from triplicates and any non-reproducible data were discarded. The relative RNA expression with differences between control and treatment being higher or equal to 2 and lower than 0.5 were considered significantly different [12].

## Results

### Effect of PGF_2α _on gene expression in primary decidual cells

**Figure 1:**
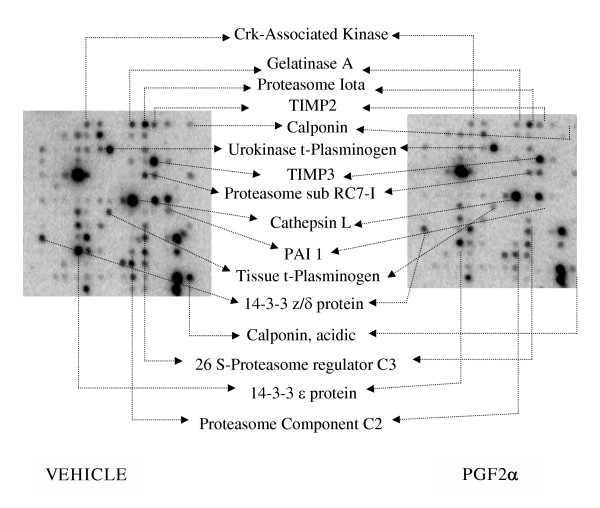
Representative cDNA expression array using mRNA obtained from rat primary decidual cells treated with either PGF_2α _or vehicle.

**Figure 2:**
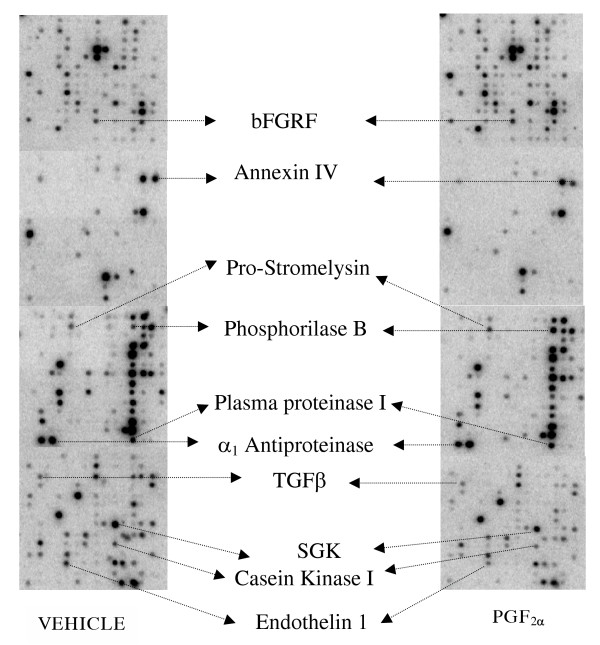
cDNA expression array of rat primary decidual cells treated with PGF_2α _or vehicle.

From the total genes available in the rat 1.2 membrane arrays, only 20% of the genes were detected in rat primary decidual cells. Sixty genes were significantly down regulated, whereas only six genes were up regulated by PGF_2α _(Figures 1 and 2). PGF_2α_-receptor gene expression was similar in both control and treated groups. On the other hand, no differences in the expression of housekeeping genes such as ribosomal proteins and β-actin could be detectedin control and PGF_2α _treated cells (data not shown).

### Effect of PGF_2α _on the expression of genes related to the extracellular matrix (ECM)

The majority of genes whose expression was affected by PGF_2α _in primary decidual cells are genes involved in the regulation of the ECM. Genes such as gelatinase A (MMP2), cathepsin L, tissue inhibitor metalloproteinases 2 (TIMP2) and 3 (TIMP3), plasminogen activator inhibitor1 (PAI1), tissue type plasminogen activator (tPA), urokinase plasminogen activator (uPA), endothelin 1, calponin, carboxypeptidase D and calponin acidic were down regulated. The opposite effect was observed for prostromelysin 53 kDa (proMMP3), plasma proteinase I alpha and alpha 1 antiproteinase – all of which were significantly up regulated by PGF_2α _(Table 1).

**Table 1:** Effect of PGF_2α _on the expression of genes related to the extracellular matrix (ECM).

**Gene Bank number**	**Swiss protein number**	**Name**	**Function**	**Ratio**	**% decrease**	**Fold increase**
U65656	P97581	Gelatinase A	Metalloproteinase	0.04 ± 0.02	87.53	
Y00697	P07154	Cathepsin L	Cysteine protease	0.09 ± 0.05	91.09	
L31884	P30121	Tissue inhibitor metalloproteinase 2 (TIMP2)	Protease inhibitor	0.05 ± 0.01	95.54	
U27201	P48032	Tissue inhibitor metalloproteinase 3 (TIMP3)	Protease inhibitor	0.04 ± 0.01	91.95	
X63434	P29598	Urokinase type Plasminogen activator (uPA)	Serine protease	0.14 ± 0.09	94.86	
M24067	P20961	Plasminogen activator inhibitor (PAI)	Protease inhibitor	0.09 ± 0.05	91.68	
M2367	P19367	Tissue type plasminogen activator (tPA)	Serine protease	0.17 ± 0.02	89.90	
X71071	Q08290	Calponin	Cytoskeleton	0.08 ± 0.004	92.50	
U06755	P37397	Calponin, acidic	Cytoskeleton	0.24 ± 0.13	76.76	
U62897	O35850	Carboxypeptidase D	Carboxypeptidase	0.24 ± 0.14	76.48	
M64711	P22388	Endothelin 1	Hormone	0.34 ± 0.2	77.48	
X02601	P03957	Polypeptide, 53 kDa, growth factor induced	Metalloproteinase	2.54 ± 1.04		3
J03552	P14046	Plasma proteinase inhibitor α1-inhibitor III	Protease inhibitor	3.64 ± 0.64		4
M32247	P17475	α1 antiproteinase	Protease inhibitor	2.45 ± 0.44		3

Ratio = net intensity of PGF_2α _normalized/net intensity of control normalized, and represent the mean ± SEM of each ratio from separate experiments. Ratio > 2 = up regulation; ratio < 0.5 = down regulation; 0.5 < Ratio < 2 = equal expression.

### PGF_2α _regulation of genes related to the proteasome protein component system

Messenger RNA encoding proteasome Iota, proteasome component C2, proteasome subunit RC7-I, 26-S proteasome regulator and proteasome component C3 were down regulated by PGF_2α_. Only proteasome component C9 was up regulated after PGF_2α _treatment (Table 2).

**Table 2:** PGF_2α _regulation of genes related to the proteasome component system.

**Gene Bank number**	**Swiss Protein number**	**Name**	**Function**	**Ratio**	**% decrease**	**Fold increase**
D10755	P34062	Proteasome Iota	Proteosomal protein	0.07 ± 0.04	93.64	
M29859	P18420	Proteasome component C2	Proteosomal protein	0.31 ± 0.15	78.28	
U77918	Q63569	26-proteasome component C3	Proteosomal protein	0.39 ± 0.08	61.94	
J02897	P17220	Proteasome component C3	Proteosomal protein	0.31 ± 0.02	59.02	
D21799	P40307	Proteasome subunit RC7-I	Proteosomal protein	0.18 ± 0.01	82.62	
X55986	P21670	Proteasome subunit C9	Proteosomal protein	2.32 ± 0.72		3

### PGF_2α _regulation of genes involved in trafficking and signal transduction

The mRNAs encoding for 14-3-3ε and z/δ proteins, and Annexin IV were down regulated in PDC after PGF_2α _treatment (Table 3). PGF_2α _down regulated the expression of serum glucocorticoid kinase (SGK), casein kinase I, extracellular signal-regulated kinase (ERK-1), male germ cell-associated kinase (MAK), and Wee tyrosine kinase. PGF_2α _also significantly inhibited Crk-associated kinase (CAS), calcium calmodulin kinase II (CAMKII) and IV (CAMKIV), phospholipase C1, and protein phosphatases such as phosphatase 2A and protein tyrosine phosphatase 1B (Table 3).

**Table 3:** PGF_2α _regulation of genes encoding proteins involved in trafficking and signal transduction.

**Gene Bank number**	**Swiss protein number**	**Name**	**Function**	**Ratio**	**% decrease**	**Fold increase**
D17615	P97286	14-3-3 z/δ protein	Traficking protein	0.14 ± 0.07	86.07	
M84416	P29360	14-3-3 Epsilon protein	Traficking protein	0.29 ± 0.15	71.63	
L01624	Q06226	Serum Glucocorticoid kinase (SGK)	Kinase	0.25 ± 0.12	93.49	
L07578	Q06486	Casein kinase I δ	Kinase	0.29 ± 0.15	72.18	
M61177	P21708	Extracellular signal-regulated kinase 1 (ERK1)	Kinase	0.29 ± 0.13	66.67	
D29766	Q63766	Crk-associated kinase	Kinase	0.09 ± 0.06	87.16	
M35862	P20793	Male germ cell-associated kinase (Mak)	Kinase	0.20 ± 0.11	78.6	
D31838	Q63802	Wee tyrosine kinase	Cell cycle kinase	0.23 ± 0.12	69.47	
M20637	P10688	Phospholipase C δ1	Phospholipase PI kinase	031 ± 0.15	68.24	
J04503	P20650	Protein phosphatase 2C α	Phosphatase	0.18 ± 0.03	58.87	
L12385	P26438	Protein Phosphatase 2A	Phosphatase	0.38 ± 0.13	65.91	
L13408	P15791	Ca^+2^/Calmodulin dependent kinase II δ subunit	Kinase	0.34 ± 0.12	72.12	
M63334	P13234	Ca^+2^/Calmodulin dependent kinase IV	Kinase	0.29 ± 0.15	70.90	
M33962	P20417	Protein Tyrosine phosphatase 1B	Phosphatase	0.21 ± 0.08	79.77	
D38224	P55260	Annexin IV (ANX4)	Exocytosis protein	0.14 ± 0.08	86.34	
L24810	Q64572	Ca^+2^/Calmodulin Phosphorylase B	Kinase	5.06 ± 2.06		5

### PGF2α effect on the expression of genes associated with G-proteins, growth factors, chemokines and cytokines

The 5-hydroxytryptamine receptor 2A (HTR2A), adenosine A2A and A2B receptors (ADORA2A and ADORA2B respectively), guanine nucleotide-binding protein G α3 subunit (GN-BPG α_3_), guanine nucleotide-binding protein α stimulating (GN-BP α stimul.), Rab 11A and guanine nucleotide-binding protein α12 subunit (GN-BP α_12_) gene expression were down regulated by PGF_2α _(Table 4). The mRNA expression of growth factors such as bone morphogenetic protein 4 (BMP4), ADP ribosylation factor 5 and 6 (ADPRF5 and 6 respectively), glia maturation factor β (GMFB), and transforming growth factor β I (TGF-βI) decreased after PGF_2α _treatment as well (Table 1). Interferon inducible protein (Table 4) and leukocyte common antigen (data not shown) were the only cytokine and chemokine related genes found significantly down regulated in response to PGF_2α_. Conversely, PGF_2α _stimulated the expression of basic fibroblast growth receptor factor 1 (bFGRF1) (Table 4).

**Table 4:** Effect of PGF_2α _on the expression of genes associated with G-proteins, growth factors, chemokines and cytokines.

**Gene Bank number**	**Swiss protein number**	**Name**	**Function**	**Ratio**	**% decrease**	**Fold change**
X52498	P17246	Transforming Growth Factor β1	Growth factor	0.33 ± 0.2	72	
M60921	P27049	Antiproliferative B-cell translocation gene 2 (BTG2)	Growth factor	0.28 ± 0.15	51	
X61381	P26376	Interferon-induced protein	Chemokine	0.25 ± 0.14	56	
L11586	Q64604	Leukocyte common antigen	Intracell. phosphatase	0.07 ± 0.02	83	
Z22607	Q06826	Bone Morphogenetic protein 4 (BMP4)	Growth factor	0.34 ± 0.17	78	
L12384	P26437	ADP Ribosylation factor 5	Intracellular transducer	0.02 ± 0.008	67	
L12385	P26438	ADP Ribosylation factor 6	Intracellular transducer	0.18 ± 0.07	83	
Z11558	Q63228	Glia maturation factor β (GMFB)	Growth factor	0.17 ± 0.07	83	
M30705	P14842	5-hydroxytryptamine receptor 2A (HTR2A)	G proteins	0.06 ± 0.03	93	
S47609	P30543	Adenosine A2A receptor (ADORA2A)	G proteins	0.13 ± 0.08	87	
M91466	P29276	Adenosine A2B receptor (ADORA2B)	G proteins	0.17 ± 0.07	83	
M20713	P08753	Guanine nucleotide-binding protein G α3 subunit	G proteins	0.22 ± 0.14	78	
M17525	P04894	Guanine nucleotide-binding protein α stimulating	G proteins	0.24 ± 0.13	75	
M75153	P24410	Rab-11A	G proteins	0.18 ± 0.11	82.	
D85760	Q63210	Guanine nucleotide-binding protein α12 subunit	G proteins	0.15 ± 0.09	85	
D12498	Q04589	Basic Fibroblast Growth Receptor factor 1	Growth factor	2.32 ± 1.07		3

## Discussion

Levels of PGF_2α _in the uterus need to be under tight control to avoid interference with the establishment and progression of pregnancy. Pathophysiological elevations in PGF_2α _lead to excessive uterine contractions. Therefore, PGF_2α _production must be avoided to maintain a quiescent uterus [13]. In this paper we present initial data demonstrating that elevated PGF_2α _can target the decidua, an endocrine tissue whose integrity is fundamental for the success of implantation and for the progression of pregnancy. Our results demonstrate that elevated PGF_2α _down regulate the expression of decidual genes related to the proteasome protein component system, those involved in trafficking and signal transduction and genes associated with G-proteins, growth factors, chemokines and cytokines. However, interestingly, the main effect of PGF_2α _is related to the turnover and degradation of the ECM which provides mechanical strength to the tissue. Thus, in addition to inducing uterine contractions, PGF_2α _can have a noxious impact on the success of implantation and progression of pregnancy, at least in part by deregulating the turnover of the ECM in the decidua.

The largest group of genes down regulated by PGF_2α _is directly or indirectly related with the turnover and degradation of the ECM. Genes such as gelatinase A (MMP-2), TIMP2 and 3 [14,15], cathepsin L [16], carboxypeptidase D are included in different groups of proteases, and can directly affect the degradation of the ECM. Carboxypeptidase D (CPD) or metallocarboxypeptidase D is a 180-kDa protein that contains almost three carboxypeptidase-like domains, a transmembrane domain, and a cytosolic tail. This gene participates in the processing of proteins transiting the secretory pathway [17]. TGF-β is a key factor that favours accumulation of collagen, laminin and fibronectin in the ECM [17]. TGF-β can stimulate PAI-1, inhibiting the protease degradation of the ECM. Other factors included in the plasminogen/plasmin and fibrinolytic system, such as tPA, uPA and PAI-1, can participate in the tissue remodelling of the ECM directly through the binding to specific receptors, and through the transformation of the plasminogen precursor bound to the cell surface to plasmin, which is an active serine protease. Plasmin is able to degrade most of the components of the ECM either directly or indirectly by the activation of MMPs. All of the plasminogen/plasmin factors mentioned previously participate in the process of decidualization [19-21].

Endothelin-1 is a peptide characterized as a potent endothelial cell-derived vasoconstrictor. It is synthesized as an inactive precursor (preproendothelin) and processed to a mature active form (endothelin) by zinc metalloproteinases. The active form, endothelin-1, promotes synthesis of collagen types I and II by fibroblasts, affects the ECM remodelling and promotes the proliferation of mesangial cells [21]. Endothelin-1 is also associated with neovascularization and regulation of blood flow [22]. This vasoactive factor is also involved in the genesis of endothelial cells behaviour [22,23].

Annexin IV (ANX4, also called Lipocortin) was differentially down regulated by PGF_2α_. This protein belongs to a family of intracellular proteins that binds membrane phospholipids in a calcium-dependent manner. Thus, it can also inhibit phospholipase A2 (PLA2) [24]. ANX4 can also directly bind glycosaminoglycan (GAG) and can be localized not only to the cytoplasm but also the cell surface or the extracellular compartment [25]. It has been proposed that ANX4 could affect the mobilization of different substrates involved in the regulation of the ECM. Moreover, Annexin IV and other annexins can act as ligands for proteoglycans localized on the cell surface, in the ECM, or in secretory granules [24,25].

Calponin (CaP) has three genetic isoforms, h_1_, h_2 _and acidic calponin, and can be identified by the individual C-terminal tail sequences. The c-terminal sequences regulate actin association and the cytoskeleton [26,27]. It is known that Cap or basic Cap inhibit the actomyosin ATPase in a calmodulin dependent manner [27]. Basic Cap, by affecting microtubules assembly, can modify the cytoskeleton of the cells, and indirectly, the associated ECM [27,28]. On the other hand, acidic Cap belongs to the family of actin-associated proteins. It can interact with F-actin but not with microtubules, desmin filaments, and tropomyosin as basic Cap does. These properties suggest that acidic Cap is functionally distinct from basic Cap and could affect the ECM in a different manner [29].

Pro-stromelysin, α_1 _antiproteinase, plasma proteinase-Iα inhibitor, basic fibroblast growth receptor factor 1, phosphorylase B and proteasome component C9 were the only genes up regulated by PGF_2α _in PDC. Pro-stromelysin is a 53 kDa peptide and a pro-MMP3 precursor and is directly related with the ECM regulation [30]. Plasma proteinase 1α inhibitor belongs to a major group of proteins that includes α2-macroglobulin [31], an important protein secreted by the decidual cells which has a critical role in the control of implantation [31]. Growth factor receptors, such as bFGRF, are part of a multigene family of structurally related factors (FGFs 1 to 9), some of which bind heparin sulphate proteoglycans that are components of the ECM [32]. bFGRFs as well as bFGF are also temporally and spatially present in the pregnant rat uterus [33-35]. Another member of the TGFβ superfamily expressed in the uterus during early implantation and down-regulated by PGF_2α _was BMP4, a gene which appears to be involved in specific stages of embryo development and is [36].

Proteasome component system proteins, such as proteasome Iota, proteasome component C2, proteasome subunit RC-7 I, 26 S-proteasome and proteasome component C3 were down regulated by PGF_2α_. Proteosomal component systems are the main non-lysosomal proteolytic structures of the cells that participate in the elimination of abnormal proteins, short half-life proteins, and proteins controlling cell cycle [37]. During the process of cell differentiation, the level of proteasome expression and its localization varies. The proteasomal proteins can be intermediaries of ECM by contributing to the modulation of the cell cycle through the induction of proteasomal degradation of cyclin dependent kinase 2. Cell attachment to ECM components, such as fibronectin (FN), does not affect p21 mRNA levels, but the stability of the p21 protein decreased [36]. Kinase activities such as ERKs, calcium/calmodulin kinase II and IV, SGK, and casein kinase can affect the ECM downstream or upstream [38]. On the other hand, ECM can regulate the availability of substrates, as well as factors or effectors downstream or upstream related with different cascades of signal transduction pathways. For example, proteins such as decorin are components of the ECM in many tissues and appear to be involved in matrix assembly [39]. Decorin can cause the rapid phosphorylation of EGF with the concurrent activation of mitogen-activated kinase protein kinase signal pathway. Via TGFβ, decorin can interact with the MAP kinases signal transduction pathways and cross talk with calcium /calmodulin-dependent kinase II [40], which in the cDNA array was down-regulated by PGF_2α_.

Crk-associated substrate (CAS) was down-regulated by PGF_2α _and is another gene related to the ECM. Active CAS can modulate changes in cell motility and gene expression by the various MAP kinase cascades, and modify the organization of the actin cytoskeleton [39]. Another gene down-regulated by PGF_2α _was SGK, which is a transcriptionally-regulated serine/threonine protein kinase with 45–55% homology to the catalytic domain of Akt/PKB protein kinase A [40]. Skg is expressed in decidual tissue and can be activated by the phosphoinositide 3-kinase pathway (PI3-Kinase) through PDK1-mediated phosphorylation [41]. Traficking proteins such as 14-3-3 z/δ and ε are involved in the regulation of genes related to the ECM because it can block the activation cascade of signal transduction pathways [42].

Protein phosphatase type 2A (PP2A) was down regulated by PGF_2α_. The first and most important point of control of PP2A is at the transcriptional level. The increase of phosphatase activity corresponded with a decrease in the phosphorylation of cellular proteins in anchorage-dependent cells, but much lesser regulated in anchorage-independent cells [43].

It is well known that members of the proinflammatory cytokine family can induce MMP expression in numerous tissues [44]. Also, cytokines and chemokines, such as interferon inducible protein and leukocyte common antigen, are associated with the local induction of MMP expression in response to proinflammatory cytokines. The indirect action of these molecules through MMPs may aid to generate different changes in the endometrial stroma during maternal recognition of pregnancy [44]

We have also compared the pattern of gene expression in rat decidual tissue *in vivo *on day 12 of pseudopregnancy (when the decidua undergoes regression) with that of the PGF_2α _treatment in rat PDC *in vitro *(data not shown). Interestingly, we found a 49% coincidence in the genes that were down regulated in both experimental situations. This coincidence suggests a relationship between the physiological regression and reorganization of the decidual tissue that occurs on day 12 of pseudopregnancy, with the pattern of gene expression in rat PDC after PGF_2α _treatment. On the other hand, many of the genes whose expression was affected by PGF_2α _in the rat PDC, are expressed in the decidua during decidualization and implantation. This suggests a possible connection between the action of PGF_2α _and the physiology of the decidual tissue. Moreover, the ECM is an important component of the decidualization and implantation process. Any modification in its turnover or degradation could affect the formation of the decidua, blastocyst invasion, and the timing of decidual regression and reorganization. It is known that the expression of MMPs/TIMPs plays an important role in the control of implantation. If PGF_2α _silences or decreases the expression of genes related with the systems aforementioned, it also could affect tissue remodelling by directly modifying the proteases involved in this process, or indirectly by affecting the ECM turnover and degradation, important in the accumulation of a spongy mass of tissue around each embryo during decidualization [45]. Most probably, many of PGF_2α_'s effects on the expression of genes involved in ECM turnover are indirect. Alterations of genes involved in different signalling pathways such as ERK-1, CaMCKII, PLCδ1 and G-protein subunits may impact the expression of a great number of transcripts.

In summary, our results show, for the first time, that pathophysiological concentrations of PGF_2α _have a severe impact on the expression of numerous genes associated with the turnover of the ECM in the rat decidua. Future investigation should corroborate the differentially regulated genes, at the level of the message, protein, and in some cases, such as for the metalloproteinases, at the level of activity of the proteins. These data will contribute to the design of future studies on a cluster of gene candidates as targets of PGF_2α _action in this endocrine tissue.

## Abbreviations

TIMP2 = tissue inhibitor metalloproteinases 2; TIMP3 = tissue inhibitor metalloproteinases 3; PAI 1 = plasminogen activator inhibitor 1; uPA = urokinase type; tPA = tissue type palsminogen activator; TGFβ = transforming growth factor β; SGK = serum glucocorticoid kinase; ERK-1 = extracellular signal-regulated kinase-1; MAK = male germ cell-associated kinase; CAS = crk-associated kinase kinase; CamKII = calcium calmodulin kinase II; CamKIV = calcium calmodulin kinase IV; PLC δ_1 _= phospholipase C delta 1; BMP 4 = bone morphogenetic factor 4; ADPRF 5 = ADP ribosylation factor 5; bFGFRF1 = basic fibroblast growth factor receptor F1; HTR2A = 5-hydroxytryptamine receptor 2A; ADORA2A = adenosine receptor A2A; ADORA2B = adenosine receptor A2B; GN-BPG α_3 _= guanine nucleotide-binding protein G α_3_; GN-BP α stimul. = guanine nucleotide-binding protein α stimulating; GN-BPG α_12 _sub. = guanine nucleotide-binding protein α_12 _subunit

## Authors' contributions

EC and SFG carried out the decidualization, and primary decidual cell culture. EC isolated the RNA isolation, performed the cDNA array assay, the analysis of the results, and drafted the manuscript. GG conceived the study and edited the manuscript. All authors read and approved the final manuscript.

**Figure 3:**
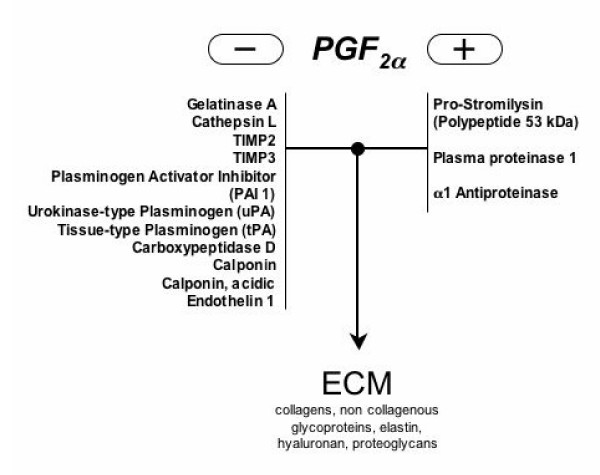
PGF_2α _has major effects on extracellular matrix (ECM) regulation
